# Supplementary material for: Analytical Validation of a Genomic Newborn Screening Workflow
Source: Int J Neonatal Screen. 2025 Oct 10;11(4):91. doi: 10.3390/ijns11040091 (PMC12551001; doi:10.3390/ijns11040091)
Supplement: Supplementary file 1 [file IJNS-11-00091-s001.zip › IJNS-3792665-supplementary.pdf]

Figure S1: Quality and size of extracted DNAs

(a)

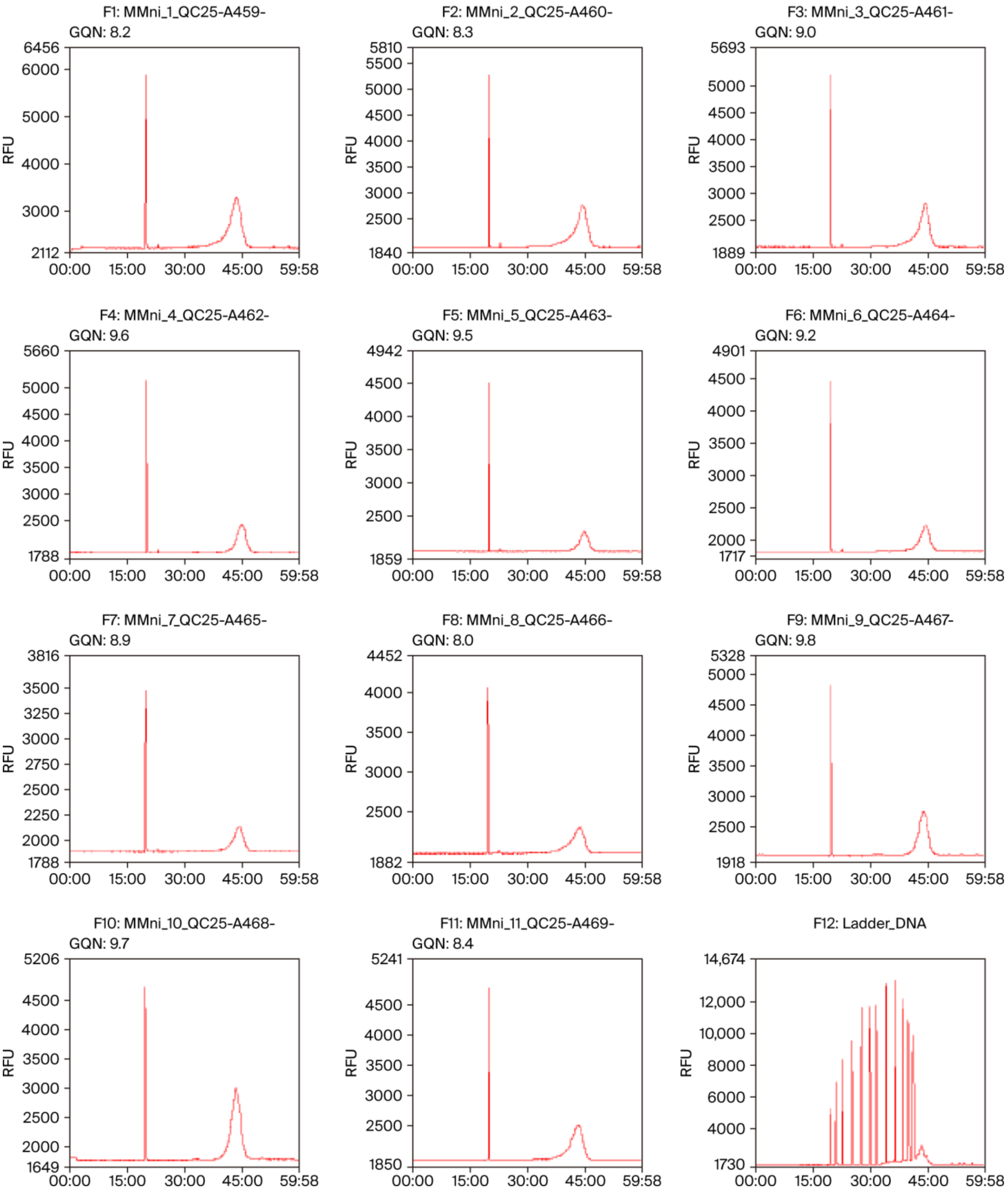

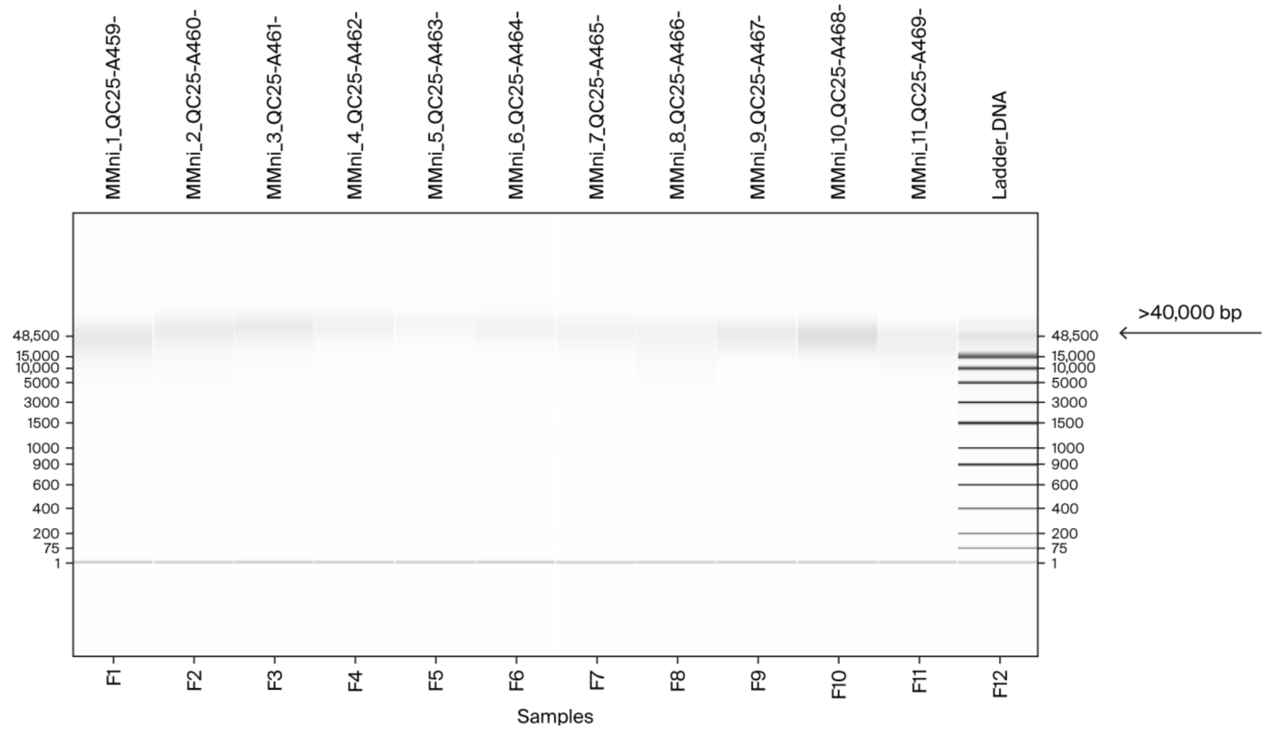

a. ***Capillary electrophoresis image****: 11 DNA samples extracted with Qiasymphony workstation (F1-F11). F12-well contains the HS extended genomic DNA ladder (5200 Fragment analyzer, DNF-364-u125, Agilent, USA).*

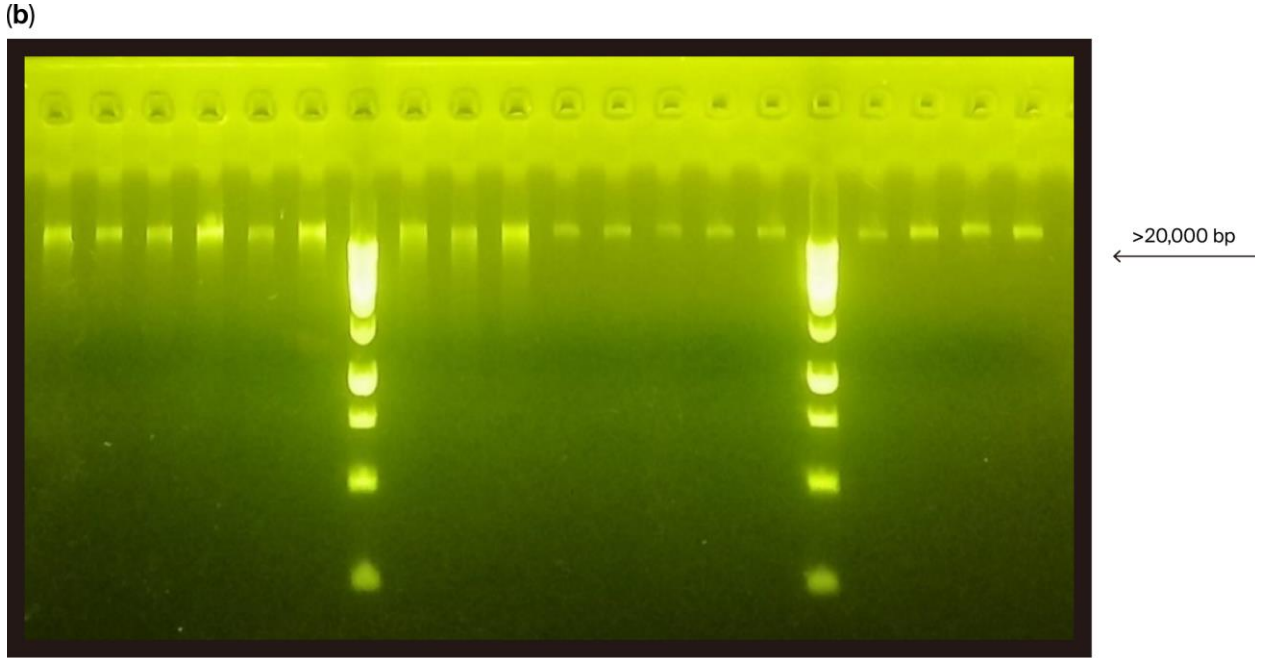

b. ***Agarose gel image****: 18 DNA samples extracted manually with QIAamp DNA Investigator Kit, loaded on 2% agarose gel. Positions on the gel are marked, and X contain sizing ladder, (Thermo Fisher scientific™ gene ruler 1 kb plus dna ladder).*

Figure S2: The quality metric thresholds for panel-v1 and panel-v2

	Panel-v1		
**Metrics**	**Threshold Low**	**Threshold High**	**Justification**
TARGET_BASES_30X_pct	93	NA	IQR × 1.5
Q30_pct	85	NA	Illumina
SNP_REFERENCE_BIAS	NA	0.56	IQR × 1.5
PF_BASES	694900749.5	NA	IQR × 1.5
MEAN_TARGET_COVERAGE	100	NA	Arbitraire
Reads_aligned_pct	99.6	NA	IQR × 1.5
SELECTED_BASES_pct	40	NA	Arbitraire
MEAN_INSERT_SIZE	150	286	Arbitraire + IQR × 1.5
TOT_Duplicates_pct	NA	25	Arbitraire
FOLD_80_BASE_PENALTY	NA	1.75	IQR × 1.5
	**Panel-v2**		
**Metrics**	**Threshold Low**	**Threshold High**	**Justification**
TARGET_BASES_30X_pct	99	NA	IQR × 1.5
Q30_pct	90	NA	IQR × 1.5
SNP_REFERENCE_BIAS	NA	0.55	IQR × 1.5
PF_BASES	2.30847×10^{11}	NA	IQR × 1.5
MEAN_TARGET_COVERAGE	117	NA	IQR × 1.5
Reads_aligned_pct	99.6	NA	IQR × 1.5
SELECTED_BASES_pct	78	NA	IQR × 1.5
MEAN_INSERT_SIZE	152	266	IQR × 1.5
TOT_Duplicates_pct	NA	27	IQR × 1.5
FOLD_80_BASE_PENALTY	NA	3.9	IQR × 1.5
VIP metrics			

Figure S3: DNA concentration of samples extracted by manual vs automated methods. (a) Concentration per individual extraction run. (b) Grouped concentration by extraction method.

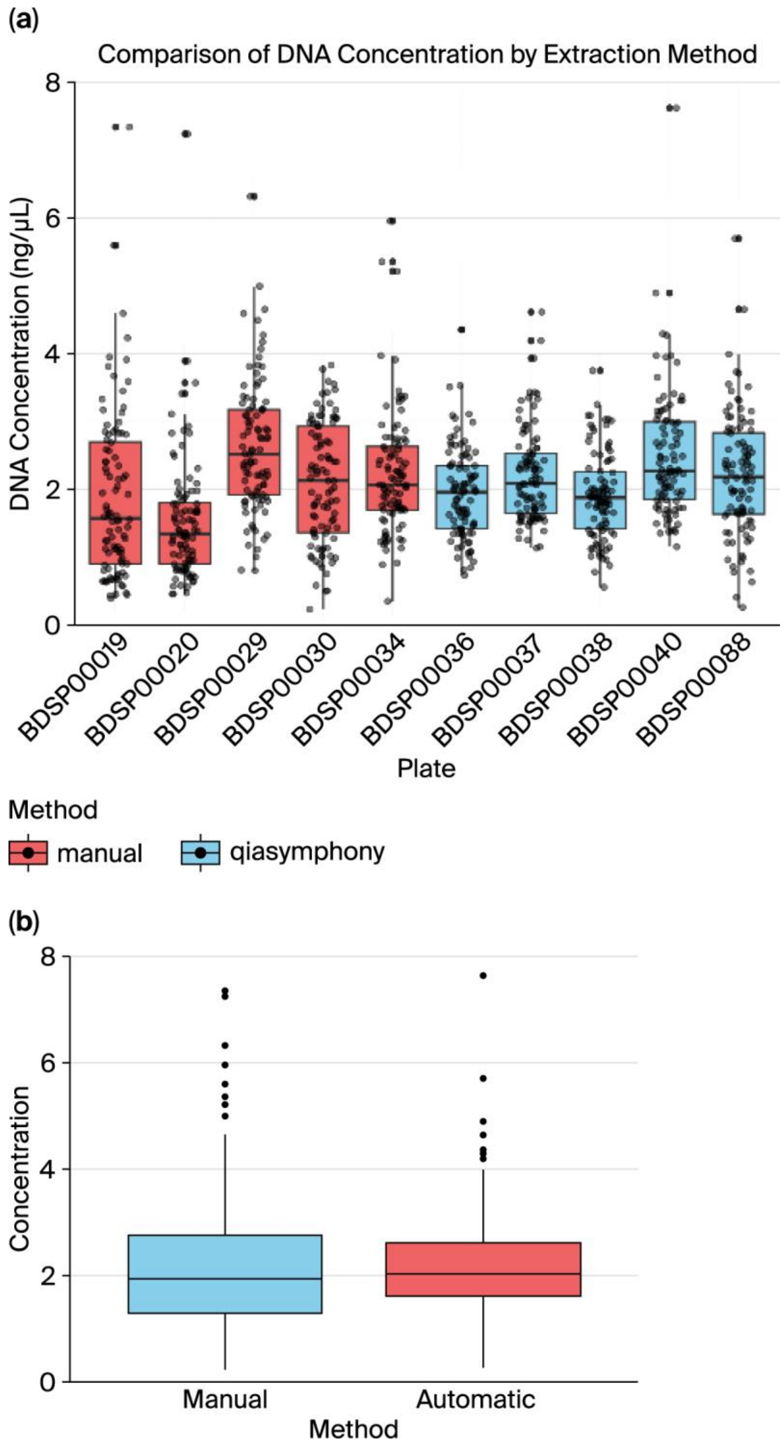

Chart S1: Levey-Jennings monitoring chart of call quality parameter for SERPINA1 c.1096 G>A of internal control sample. *Standard deviation (σ) thresholds were calculated based on median call quality of 7 consecutive runs. Call quality of 7 additional runs is plotted on the graph.*

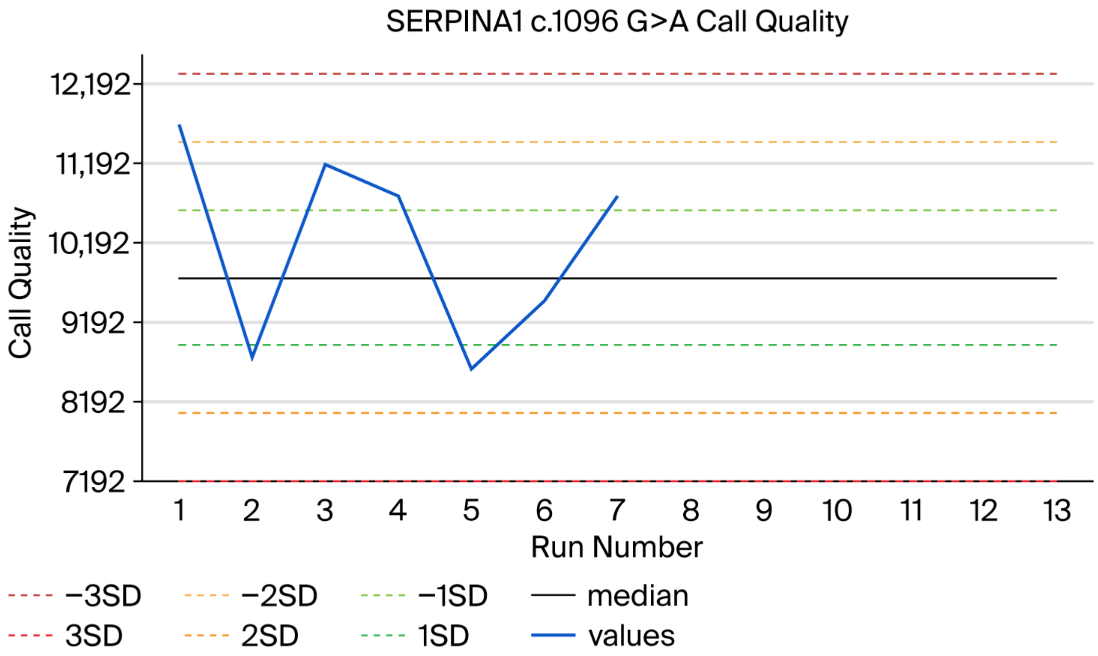

Table S1. Sample layout of the validation plates used for sequencing method evaluation.

ID of samples

	1	2	3	4	5	6	7	8	9	10	11	12
A	NBPOS-1.1	NBPOS-1.2	NBPOS-1.3	HG002.1_NA24385	NBNEG-1.1	NBNEG-1.2	NBNEG-1.3	ADNEG-1.1	ADNEG-1.2	ADNEG-1.1	ADNEG-1.2	/
B	NBPOS-2.1	NBPOS-2.2	NBPOS-2.3	HG002.2_NA24385	NBNEG-2.1	NBNEG-2.2	NBNEG-2.3	ADNEG-2.1	ADNEG-2.2	ADNEG-2.1	ADNEG-2.2	/
C	NBPOS-3.1	NBPOS-3.2	NBPOS-3.3	HG002.3_NA24385	NBNEG-3.1	NBNEG-3.2	NBNEG-3.3	ADNEG-3.1	ADNEG-3.2	ADNEG-3.1	ADNEG-3.2	/
D	NBPOS-4.1	NBPOS-4.2	NBPOS-4.3	HG002.4_NA24385	NBNEG-4.1	NBNEG-4.2	NBNEG-4.3	ADNEG-4.1	ADNEG-4.2	ADNEG-4.1	ADNEG-4.2	/
E	NBPOS-5.1	NBPOS-5.2	NBPOS-5.3	HG002.5_NA24385	NBNEG-5.1	NBNEG-5.2	NBNEG-5.3	DBS-ADNEG-1.1	DBS-ADNEG-1.2	DBS-ADNEG-1.1	DBS-ADNEG-1.2	/
F	NBPOS-6.1	NBPOS-6.2	NBPOS-6.3	HG002.6_NA24385	NBNEG-6.1	NBNEG-6.2	NBNEG-6.3	DBS-ADNEG-2.1	DBS-ADNEG-2.2	DBS-ADNEG-2.1	DBS-ADNEG-2.2	/
G	NBPOS-7.1	NBPOS-7.2	NBPOS-7.3	HG002.7_NA24385	NBNEG-7.1	NBNEG-7.2	NBNEG-7.3	DBS-ADNEG-3.1	DBS-ADNEG-3.2	DBS-ADNEG-3.1	DBS-ADNEG-3.2	/
H	NBPOS-8.1	NBPOS-8.2	NBPOS-8.3	HG002.8_NA24385	NBNEG-8.1	NBNEG-8.2	NBNEG-8.3	DBS-ADNEG-4.1	DBS-ADNEG-4.2	DBS-ADNEG-4.1	DBS-ADNEG-4.2	/

a. NovaSeq plate

ID of samples

	1	2	3	4	5	6	7	8	9	10	11	12
A	NBPOS-1.1	NBPOS-1.2	HG002.1_NA24385	NBNEG-1.1	NBNEG-1.2	ADNEG-1.1	/	/	/	/	/	/
B	NBPOS-2.1	NBPOS-2.2	HG002.2_NA24385	NBNEG-2.1	NBNEG-2.2	ADNEG-2.1	/	/	/	/	/	/
C	NBPOS-3.1	NBPOS-3.2	HG002.3_NA24385	NBNEG-3.1	NBNEG-3.2	ADNEG-3.1	/	/	/	/	/	/
D	NBPOS-4.1	NBPOS-4.2	HG002.4_NA24385	NBNEG-4.1	NBNEG-4.2	ADNEG-4.1	/	/	/	/	/	/
E	NBPOS-5.1	NBPOS-5.2	HG002.5_NA24385	NBNEG-5.1	NBNEG-5.2	DBS-ADNEG-1.1	/	/	/	/	/	/
F	NBPOS-6.1	NBPOS-6.2	HG002.6_NA24385	NBNEG-6.1	NBNEG-6.2	DBS-ADNEG-2.1	/	/	/	/	/	/
G	NBPOS-7.1	NBPOS-7.2	HG002.7_NA24385	NBNEG-7.1	NBNEG-7.2	DBS-ADNEG-3.1	/	/	/	/	/	/
H	NBPOS-8.1	NBPOS-8.2	HG002.8_NA24385	NBNEG-8.1	NBNEG-8.2	DBS-ADNEG-4.1	/	/	/	/	/	/

b. NextSeq plate

Two microtiter plates were designed to assess sequencing performance on different platforms: (a) a NovaSeq plate containing 88 samples and (b) a NextSeq plate with 48 samples. Both plates include DNA samples from newborn dried blood spots (DBS), adult whole blood, and the HG002 Genome in a Bottle (GIAB) reference material. Sample types include positive and negative newborn samples, negative adult controls (both in whole blood and DBS format), and are distributed in multiplexed configurations (3-plex, 4-plex, 8-plex).

Table S2: Concordance calculation between manual vs automated extractions for workflow of panel-v2

Run replicate 1 (manual)	Replicate 1 (A)	Run replicate 2 (qiasymphony)	Replicate 2 (B)	variants in both replicates (C)	only in replicate 1 (A)	only in replicate 2 (B)	Concordance
Panel_V2_12	Sample 1a	Panel_V2_05	Sample 1b	1323	86	63	0,90
Panel_V2_12	Sample 2a	Panel_V2_05	Sample 2b	1422	70	78	0,91
Panel_V2_12	Sample 3a	Panel_V2_05	Sample 3b	1386	66	42	0,93
Panel_V2_12	Sample 4a	Panel_V2_05	Sample 4b	1378	74	60	0,91
Panel_V2_20	Sample 5a	Panel_V2_23	Sample 5b	1377	48	55	0,93

Table S3 : BabyDetect genes selected by Gene Inclusion Criteria

Clinical category	Gene	Clinical category	Gene	Clinical category	Gene
Cardiology	*ANK2*	Endocrinology	*POR*	Hematology	*DKC1*
Cardiology	*CACNA1C*	Endocrinology	*POU1F1* [a]	Hematology	*ERCC4*
Cardiology	*CALM1*	Endocrinology	*PROP1* [a]	Hematology	*F13A1*
Cardiology	*CALM2*	Endocrinology	*PTF1A*	Hematology	*F13B*
Cardiology	*CALM3*	Endocrinology	*SECISBP2*	Hematology	*F2*
Cardiology	*CASQ2*	Endocrinology	*SLC26A4*	Hematology	*F8*
Cardiology	*KCNE1*	Endocrinology	*SLC5A5*	Hematology	*F9*
Cardiology	*KCNJ2*	Endocrinology	*STARa*	Hematology	*FANCA*
Cardiology	*KCNQ1*	Endocrinology	*TBX19*	Hematology	*FANCB*
Cardiology	*MYBPC3*	Endocrinology	*TG*	Hematology	*FANCC*
Cardiology	*MYH7*	Endocrinology	*THRA*	Hematology	*FANCG* [a]
Cardiology	*RYR2*	Endocrinology	*THRB*	Hematology	*FANCD2*
Cardiology	*TECRL*	Endocrinology	*TNFRSF11A*	Hematology	*FANCE*
Cardiology	*TRDN*	Endocrinology	*TPO*	Hematology	*FANCF*
Endocrinology	*ABCC8* [a]	Endocrinology	*TSHR*	Hematology	*FANCI*
Endocrinology	*ALPL*	Endocrinology	*TUBB1*	Hematology	*FANCL*
Endocrinology	*CACNA1D*	Endocrinology	*UBR1*	Hematology	*FANG*
Endocrinology	*CLCN7*	Endocrinology	*UCP2* [a]	Hematology	*G6PD*
Endocrinology	*CYP11A1*	Gastroenterology	*ABCB11*	Hematology	*GATA1*
Endocrinology	*CYP11B1*	Gastroenterology	*ABCB4*	Hematology	*HBA1*
Endocrinology	*CYP11B2*	Gastroenterology	*AMN*	Hematology	*HBA2*
Endocrinology	*CYP17A1*	Gastroenterology	*APOA5*	Hematology	*HBB*
Endocrinology	*CYP21A2*	Gastroenterology	*APOC2*	Hematology	*HK1* [a]
Endocrinology	*DUOX1*	Gastroenterology	*ATP7B*	Hematology	*ITGB2*
Endocrinology	*DUOX2*	Gastroenterology	*ATP8B1*	Hematology	*MAD2L2*
Endocrinology	*DUOXA1*	Gastroenterology	*CBLIF*	Hematology	*MYSM1*
Endocrinology	*DUOXA2*	Gastroenterology	*CUBN*	Hematology	*PKLR*
Endocrinology	*FOXE1*	Gastroenterology	*DGAT1*	Hematology	*RFWD3*
Endocrinology	*FOXI1*	Gastroenterology	*DNAJC21*	Hematology	*RPL11*
Endocrinology	*GLIS3*	Gastroenterology	*EFL1*	Hematology	*RPL15*
Endocrinology	*GLUD1*	Gastroenterology	*GPIHBP1*	Hematology	*RPL18*
Endocrinology	*GNAS*	Gastroenterology	*IL10RA*	Hematology	*RPL26*
Endocrinology	*HHEX*	Gastroenterology	*IL10RB*	Hematology	*RPL27*
Endocrinology	*HNF1A* [a]	Gastroenterology	*LIPA*	Hematology	*RPL31*
Endocrinology	*HNF4A* [a]	Gastroenterology	*LMF1*	Hematology	*RPL35*
Endocrinology	*HSD3B2*	Gastroenterology	*LPL*	Hematology	*RPL35A*
Endocrinology	*INSR* [a]	Gastroenterology	*MYO5B*	Hematology	*RPL5*
Endocrinology	*IYDa*	Gastroenterology	*NEUROG3*	Hematology	*RPL9*
Endocrinology	*KAT6B*	Gastroenterology	*NR1H4*	Hematology	*RPS10*
Endocrinology	*KCNJ10*	Gastroenterology	*SERPINA1*	Hematology	*RPS15A*
Endocrinology	*KCNJ11* [a]	Gastroenterology	*SLC26A3*	Hematology	*RPS17*
Endocrinology	*MRAP*	Gastroenterology	*SLC9A3*	Hematology	*RPS19*
Endocrinology	*NFKB2*	Gastroenterology	*TJP2*	Hematology	*RPS24*
Endocrinology	*NKX2-1*	Gastroenterology	*UGT1A1*	Hematology	*RPS26*
Endocrinology	*NKX2-5*	Hematology	*ADAMTS13*	Hematology	*RPS27*
Endocrinology	*NNT*	Hematology	*BRCA2*	Hematology	*RPS28*
Endocrinology	*PAX8*	Hematology	*BRIP1*	Hematology	*RPS29*

Clinical category	Gene	Clinical category	Gene	Clinical category	Gene
Hematology	*RPS7*	Immunology	*NCF2*	Metabolic disorders	*CPT1A*
Hematology	*SBDS*	Immunology	*NCF4*	Metabolic disorders	*CPT2*
Hematology	*SH2D1A*	Immunology	*PGM3*	Metabolic disorders	*DBT*
Hematology	*SLX4*	Immunology	*PIK3R1*	Metabolic disorders	*DLD*
Hematology	*TSR2*	Immunology	*PRF1*	Metabolic disorders	*DNAJC12*
Hematology	*UBE2T*	Immunology	*PRKDC*	Metabolic disorders	*ETFA*
Hematology	*WAS*	Immunology	*PTPRC*	Metabolic disorders	*ETFB*
Hematology	*WIPF1*	Immunology	*RAB27A*	Metabolic disorders	*ETFDH*
Hematology	*XIAP*	Immunology	*RAC2*	Metabolic disorders	*FAH*
Immunology	*ADA*	Immunology	*RAG1*	Metabolic disorders	*FBP1*
Immunology	*AK2*	Immunology	*RAG2*	Metabolic disorders	*FLAD1*
Immunology	*ARPC1B*	Immunology	*RFX5*	Metabolic disorders	*G6PC*
Immunology	*BTK*	Immunology	*RFXANK*	Metabolic disorders	*GAA*
Immunology	*CD247*	Immunology	*RFXAP*	Metabolic disorders	*GALE*
Immunology	*CD3D*	Immunology	*SRP54*	Metabolic disorders	*GALK1*
Immunology	*CD3E*	Immunology	*STX11*	Metabolic disorders	*GALM*
Immunology	*CD3G*	Immunology	*STXBP2*	Metabolic disorders	*GALNS*
Immunology	*CIITA*	Immunology	*UNC13D*	Metabolic disorders	*GALT*
Immunology	*CORO1A*	Immunology	*VPS45*	Metabolic disorders	*GAMT*
Immunology	*CSF3R*	Immunology	*ZAP70*	Metabolic disorders	*GATM*
Immunology	*CTPS1*	Metabolic disorders	*ABCD4*	Metabolic disorders	*GBA*
Immunology	*CXCR4*	Metabolic disorders	*ACAD8*	Metabolic disorders	*GCDH*
Immunology	*CYBA*	Metabolic disorders	*ACAD9*	Metabolic disorders	*GCH1*
Immunology	*CYBB*	Metabolic disorders	*ACADM*	Endocrinology	*GCK*[a]
Immunology	*DCLRE1C*	Metabolic disorders	*ACADS*	Metabolic disorders	*GLDC*
Immunology	*DOCK2*	Metabolic disorders	*ACADSB*	Metabolic disorders	*GUSB*
Immunology	*DOCK8*	Metabolic disorders	*ACADVL*	Metabolic disorders	*GYS1*
Immunology	*ELANE*	Metabolic disorders	*ACAT1*	Metabolic disorders	*GYS2*
Immunology	*FAS*	Metabolic disorders	*ACSF3*	Metabolic disorders	*HADH*
Immunology	*FOXN1*	Metabolic disorders	*ADK*	Metabolic disorders	*HADHA*
Immunology	*FOXP3*	Metabolic disorders	*AGL*	Metabolic disorders	*HADHB*
Immunology	*G6PC3*	Metabolic disorders	*AHCY*	Metabolic disorders	*HCFC1*
Immunology	*GFI1*	Metabolic disorders	*ALDH7A1*	Metabolic disorders	*HGSNAT*
Immunology	*HAX1*	Metabolic disorders	*ALDOB*	Metabolic disorders	*HLCS*
Immunology	*IL2RB*	Metabolic disorders	*AMT*	Metabolic disorders	*HMGCL*
Immunology	*IL2RG*	Metabolic disorders	*ARG1*	Metabolic disorders	*HMGCS2*
Immunology	*IL7R*	Metabolic disorders	*ARSB*	Metabolic disorders	*HPD*
Immunology	*IRAK4*	Metabolic disorders	*ASL*	Metabolic disorders	*IDS*
Immunology	*JAGN1*	Metabolic disorders	*ASS1*	Metabolic disorders	*IDUA*
Immunology	*JAK3*	Metabolic disorders	*BCKDHA*	Metabolic disorders	*IVD*
Immunology	*LAT*	Metabolic disorders	*BCKDHB*	Metabolic disorders	*LMBRD1*
Immunology	*LCK*	Metabolic disorders	*BCKDK*	Metabolic disorders	*MAT1A*
Immunology	*LIG4*	Metabolic disorders	*BTD*	Metabolic disorders	*MCCC1*
Immunology	*LYST*	Metabolic disorders	*CA5A*	Metabolic disorders	*MCCC2*
Immunology	*MYO5A*	Metabolic disorders	*CBS*	Metabolic disorders	*MCEE*
Immunology	*NBN*	Metabolic disorders	*CFTR*	Metabolic disorders	*MLYCD*
Immunology	*NCF1*	Metabolic disorders	*CPS1*	Metabolic disorders	*MMAA*

Clinical category	Gene	Clinical category	Gene	Clinical category	Gene
Metabolic disorders	*MMAB*	Metabolic disorders	*SLC7A7*	Neurology	*GOT2*
Metabolic disorders	*MMACHC*	Metabolic disorders	*SMPD1*	Neurology	*GRIN2B*
Metabolic disorders	*MMADHC*	Metabolic disorders	*TAT*	Neurology	*LAMA5*
Metabolic disorders	*MMUT*	Metabolic disorders	*TCN2*	Neurology	*LRP4*
Metabolic disorders	*MOCS1*	Nephrology	*AGXT*	Neurology	*MACF1*
Metabolic disorders	*MTHFR*	Nephrology	*AQP2*	Neurology	*MUSK*
Metabolic disorders	*MTR*	Nephrology	*AVPR2*	Neurology	*MYO9A*
Metabolic disorders	*MTRR*	Nephrology	*COL4A3*	Neurology	*ODC1*
Metabolic disorders	*MVK*	Nephrology	*COL4A4*	Neurology	*PDXK*
Metabolic disorders	*NADK2*	Nephrology	*COL4A5*	Neurology	*PLEC*
Metabolic disorders	*NAGLU*	Nephrology	*CTNS*	Neurology	*PREPL*
Metabolic disorders	*NAGS*	Nephrology	*CUL3*	Neurology	*RAPSN*
Metabolic disorders	*OTC*	Nephrology	*GRHPR*	Neurology	*RPH3A*
Metabolic disorders	*OXCT1*	Nephrology	*HOGA1*	Neurology	*SLC18A2*
Metabolic disorders	*PAH*	Nephrology	*KLHL3*	Neurology	*SLC18A3*
Metabolic disorders	*PCBD1*	Nephrology	*NPHS1*	Neurology	*SLC19A2*
Metabolic disorders	*PCCA*	Nephrology	*NR3C2*	Neurology	*SLC19A3*
Metabolic disorders	*PCCB*	Nephrology	*PHEX*	Neurology	*SLC25A1*
Metabolic disorders	*PGM1* [a]	Nephrology	*SCNN1A*	Neurology	*SLC25A19*
Metabolic disorders	*PHGDH*	Nephrology	*SCNN1B*	Neurology	*SLC2A1*
Metabolic disorders	*PHKA2*	Nephrology	*SCNN1G*	Neurology	*SLC5A7*
Metabolic disorders	*PHKB*	Nephrology	*WNK1*	Neurology	*SLC6A5*
Metabolic disorders	*PHKG2*	Nephrology	*WNK4*	Neurology	*SMN1* [b]
Metabolic disorders	*PLPBP*	Neurology	*ABCD1*	Neurology	*SMN2* [b]
Metabolic disorders	*PNPO*	Neurology	*ACHRE*	Neurology	*SNAP25*
Metabolic disorders	*PSAT1*	Neurology	*AGRN*	Neurology	*SPR*
Metabolic disorders	*PSPH*	Neurology	*ALG14*	Neurology	*SYT2*
Metabolic disorders	*PTS*	Neurology	*ALG2*	Neurology	*TH*
Metabolic disorders	*PYGL*	Neurology	*ARSA*	Neurology	*TPK1*
Metabolic disorders	*QDPR*	Neurology	*ATAD1*	Neurology	*TPP1*
Metabolic disorders	*SGSH*	Neurology	*ATP7A*	Neurology	*TTPA*
Metabolic disorders	*SI*	Neurology	*CHAT*	Neurology	*UNC13A*
Metabolic disorders	*SLC16A1* [a]	Neurology	*CHRNA1*	Neurology	*VAMP1*
Metabolic disorders	*SLC1A4*	Neurology	*CHRNB1*	Oncology	*RB1*
Metabolic disorders	*SLC22A5*	Neurology	*CHRND*	Ophthalmology	*CYP1B1*
Metabolic disorders	*SLC25A13*	Neurology	*COL13A1*	Ophthalmology	*FZD4*
Metabolic disorders	*SLC25A15*	Neurology	*COLQ*	Ophthalmology	*KIF11*
Metabolic disorders	*SLC25A20*	Neurology	*DBH*	Ophthalmology	*LRP5*
Metabolic disorders	*SLC25A32*	Neurology	*DDC*	Ophthalmology	*LTBP2*
Metabolic disorders	*SLC2A2*	Neurology	*DHCR7*	Ophthalmology	*NDP*
Metabolic disorders	*SLC37A4*	Neurology	*DOK7*	Ophthalmology	*RCBTB1*
Metabolic disorders	*SLC39A8*	Neurology	*DPAGT1*	Ophthalmology	*RPE65*
Metabolic disorders	*SLC52A1*	Neurology	*FOLR1*	Ophthalmology	*TSPAN12*
Metabolic disorders	*SLC52A2*	Neurology	*GFPT1*	Ophthalmology	*ZNF408*
Metabolic disorders	*SLC52A3*	Neurology	*GLRA1*		
Metabolic disorders	*SLC5A1*	Neurology	*GLRB*		
Metabolic disorders	*SLC6A8*	Neurology	*GMPPB*		

Genes highlighted in yellow were excluded by expert from panel-v1 : tagged with « a » were removed because have low penetrance mutations and mutations causing mild form of disease, tagged with « b » were removed because of technical limitation. Genes highlighted in green were included by expert in panel-v2 after second gene curation. ***Gene Inclusion Criteria are*** *: early onset of disease (before age of 5), severe disease/ disability if not treated, available treatment, benefit from treatment, pathogenic mutation, phenotype-genotype correlation and standard of care.*

Table S4. Quality control parameters for longitudinal monitoring and their description

Sequencing monitoring	
**Parameter**	**Description**
**Q30_pct**	The percentage of bases with a quality score of 30 or higher. This parameter ensures the sequencing data quality for each sample.
**PF_BASES**	Total number of bases. This parameter verifies that the desired amount of raw sequencing data has been obtained for each sample and assesses library balance during demultiplexing.
**TOT_Duplicates_pct**	The percentage of duplicated reads (PCR or optical duplicates). Duplicated reads, being redundant (non-independent artifact observations), are excluded during variant inference, representing a loss of raw data.
**Library and capture monitoring**	
**Parameter**	**Description**
**TARGET_BASES_30X_pct:**	Percentage of targeted bases that reached a minimum effective coverage of 30X in the analysis. This parameter determines the proportion of targeted regions from which diagnostically relevant variants can be inferred.
**MEAN_TARGET_COVERAGE**	Average effective coverage of sequencing across the regions of interest. This parameter assesses data quality for subsequent variant inference.
**SELECTED_BASES_pct**	Percentage of aligned bases located on/near the captured targeted regions. This parameter, calculated before applying filters used in inference (duplication, overlap clipping, mapping quality, etc.), evaluates capture efficiency during library preparation.
**MEAN_INSERT_SIZE**	Average insert size. This parameter allows to verify that the size of the DNA fragments is as expected.
**FOLD_80_BASE_PENALTY:**	Resequencing rate required for 80% of targeted bases to achieve the average coverage of target. This measure reflects the uniformity of the coverage of targeted region.
**Alignment monitoring**	
**Parameter**	**Description**
**Reads_aligned_pct**	Percentage of reads aligned to the reference genome. This metric confirms the success of the alignment step.
**Variant Inference monitoring**	
**Parameter**	**Description**
**SNP_REFERENCE_BIAS:**	Average fraction of reads where the reference allele is observed at sites with a heterozygous single nucleotide polymorphism. This parameter reflects the slight bias associated with capture. Upward deviations can also be indicative of cross-sample contamination.

Table S5. Positive samples used in analytical validation.

	sample ID	*disease*	*gene*	*variant 1*	*variant 2*	*method of confirmation*
Validation panel-v1	**NBPOS-1**	**Phenylketonuria (PKU)**	***PAH***	**c.1066-11G>A**	**c.1315+1G>A**	**Conventional NBS and panel-sequencing**
	**NBPOS-2**	**Phenylketonuria (PKU)**	***PAH***	**c.1169A>G**	**c.898G>T**	**Conventional NBS and panel-sequencing**
	**NBPOS-3**	**Aromatic l-amino acid decarboxylase (AADCD)**	***DDC***	**c.823G>A**	**c.1037A>G**	**WGS and biochemical testing**
	**NBPOS-4**	**Cystic fibrosis (CF)**	***CFTR***	**c.1521_1523delCTT**	**c.1521_1523delCTT**	**Conventional NBS and phenotyping *CFTR***
	**NBPOS-5**	**Medium-Chain-Acyl-CoA-Déshydrogénase (MCAD)**	***ACADM***	**c.948+2T>C**	**c.1045-2A>C**	**Sanger Sequencing**
	**NBPOS-6**	**Glucose-6-phosphate dehydrogenase deficiency (G6PD)**	***G6PD***	**c.466A>G**	**c.292G>A**	**Conventional NBS and panel-sequencing**
	**NBPOS-7**	**Medium-Chain-Acyl-CoA-Déshydrogénase (MCAD)**	***ACADM***	**c.1084A>G**	**c.1084A>G**	**Conventional NBS and phenotyping *ACADM***
	**NBPOS-8**	**Cystic fibrosis (CF)**	***CFTR***	**c.3752G>A**	**c.3752G>A**	**Conventional NBS and phenotyping *CFTR***
Validation panel-v2	**NBPOS-9**	**Hemophilia B**	***F9***	**c.1024A>G**		**panel-sequencing**
	**NBPOS-10**	**Short-chain acyl-CoA dehydrogenase (SCAD) deficiency**	***ACADS***	**c.1147C>T**	**c.596C>T**	**panel-sequencing**
	**NBPOS-11**	**Glucose-6-phosphate dehydrogenase (G6PD) deficiency**	***G6PD***	**c.466A>G**	**c.292G>A**	**panel-sequencing**
	**NBPOS-12**	**Cystic fibrosis**	***CFTR***	**c.1865G>A**	**c.1865G>A**	**panel-sequencing**
	**NBPOS-13**	**Cystic fibrosis**	***CFTR***	**c.1397C>G**	**c.3209G>A**	**panel-sequencing**
	**NBPOS-14**	**Wilson disease**	***ATP7B***	**c.3207C>A**	**c.1877G>C**	**panel-sequencing**
	**NBPOS-15**	**glucose-6-phosphate dehydrogenase (G6PD) deficiency**	***G6PD***	**c.1437G>C**		**panel-sequencing**
	**NBPOS-16**	**very long-chain acyl-CoA dehydrogenase (VLCAD) deficiency**	***ACADVL***	**c.325G>A**	**c.601_603delGAG**	**panel-sequencing**

Table S6: False positive Indel variants identified after sequencing

Chromosome	Gene	Position	Variant Location	Reference in databases	Type of Variant	Reference Allele	Alternative Allele	Alternative Allele Frequency	Clinical Impact
chr1	*MACF1*	39900058	Exon-intron boundary-T rich	rs71798934: benign variant	Deletion	CT	C	0.41	eliminated by variant filtering tree
chr1	*AGL*	100366446	Exon-intron boundary	.	Insertion	G	GT	0.11	eliminated by variant filtering tree
chr1	*TBX19*	168262522	Exon-intron boundary	.	Deletion	AGT	A	0.14	eliminated by variant filtering tree
chr1	*SLC19A2*	169447008	Exon-intron boundary	.	Insertion	G	GA	0.15	eliminated by variant filtering tree
chr1	*F13B*	197009631	Exon-intron boundary	.	Insertion	C	CA	0.15	eliminated by variant filtering tree
chr1	*PTPRC*	198711170	Exon-intron boundary	.	Deletion	CA	C	0.13	eliminated by variant filtering tree
chr1	*LYST*	235826382	Exon-intron boundary	.	Deletion	TAA	T/TA	0.13/0.81	eliminated by variant filtering tree
chr1	*MTR*	237060945	End of exon 33-CT repeats	.	Deletion	CT*	C	0.25	eliminated by variant filtering tree
chr1	*RYR2*	237863790	Exon-intron boundary	.	Deletion	GT	G	0.17	eliminated by variant filtering tree
chr1	*RYR2*	237955649	Exon-intron boundary	.	Deletion	CTG*	C	0.79	eliminated by variant filtering tree
chr1	*RYR2*	237965133	Exon-intron boundary	.	Deletion	AT*	A	0.18	eliminated by variant filtering tree
chr2	*PREPL*	44569739	Exon-intron boundary	.	Insertion	T	TA	0.64	eliminated by variant filtering tree
chr2	*CALM2*	47387989	Exon-intron boundary	.	Deletion	CAAAA	C/CA	0.4/0.5	eliminated by variant filtering tree
chr2	*CHRNA1*	175614908	Exon-intron boundary	.	Deletion	CAAA	C/CA	0.19/0.76	eliminated by variant filtering tree
chr2	*CPS1*	211444533	Exon-intron boundary	.	Deletion	GGT	G	0.12	eliminated by variant filtering tree
chr2	*CPS1*	211521227	Exon-intron boundary	.	Deletion	CT	C	0.76	eliminated by variant filtering tree
chr2	*COL4A3*	228159660	Exon-intron boundary	.	Deletion	GT	G	0.76	eliminated by variant filtering tree
chr3	*FANCD2*	10088407	Exon-intron boundary	.	Deletion	AG	A	0.31	eliminated by variant filtering tree
chr3	*FANCD2*	10088409	Exon-intron boundary	rs375350046: benign variant	Deletion	TAAG	T	0.3	eliminated by variant filtering tree
chr3	*FANCD2*	10089549	Exon-intron boundary	rs113574694: no clinical significance	Deletion	TACTA*	T	0.3	eliminated by variant filtering tree
chr3	*SI*	164776913	Exon-intron boundary	rs60170840: benign variant	Insertion	C	CAT	0.3	eliminated by variant filtering tree
chr4	*IDUA*	996501	Exon-intron boundary	rs150523349: benign variant	Insertion	G*	GC	1	eliminated by variant filtering tree
chr4	*ANK2*	114176848	Exon-intron boundary	rs34725737: benign variant	Insertion	G	GA	0.13	eliminated by variant filtering tree
chr4	*ETFDH*	159618695	Exon-intron boundary	.	Deletion	CT	C	0.18	eliminated by variant filtering tree

chr5	*PIK3R1*	67576330	Exon-intron boundary	.	Deletion	CT	C	0.24	eliminated by variant filtering tree
chr5	*ALDH7A1*	125929000	Exon-intron boundary	.	Deletion	AAC*	A	0.23	eliminated by variant filtering tree
chr5	*KLHL3*	137013350	Exon-intron boundary	.	Deletion	CA	C	0.24	eliminated by variant filtering tree
chr6	*BCKDHB*	80910622	Exon-intron boundary	.	Deletion	CT	C	0.14	eliminated by variant filtering tree
chr6	*TRDN*	123869769	Exon-intron boundary -A rich	.	Deletion	TAAA*	T/TA	0.16/0.83	eliminated by variant filtering tree
chr7	*PSPH*	56085117	Exon-intron boundary -A rich	.	Insertion	G	GA	0.24	eliminated by variant filtering tree
chr7	*SLC25A13*	95775848	Exon-intron boundary -A rich	.	Deletion	TA*	T	0.15	eliminated by variant filtering tree
chr8	*HGSNAT*	43002052	Exon-intron boundary-T rich	.	Deletion	CT*	C	0.32	eliminated by variant filtering tree
chr8	*PRKDC*	48805816	exon 32	rs11411516: benign variant	Insertion	A	AG	1	eliminated by variant filtering tree
chr8	*TG*	133900840	Exon-intron boundary	.	Insertion	A	ATG	0.79	eliminated by variant filtering tree
chr9	*DOCK8*	432140	Exon-intron boundary-T rich	.	Deletion	CTTT	C/CT	0.16/0.82	eliminated by variant filtering tree
chr9	*GRHPR*	37436632	Exon-intron boundary	.	Insertion	C	CCT	0.87	eliminated by variant filtering tree
chr9	*TJP2*	71849314	Exon-intron boundary	rs202100183: benign variant	Deletion	CT	C	0.18	eliminated by variant filtering tree
chr10	*COL13A1*	71647196	Exon-intron boundary-T rich	.	Insertion	G	GT	0.19	eliminated by variant filtering tree
chr10	*ADK*	76349020	Exon-intron boundary-T rich	.	Deletion	CT*	C	0.17	eliminated by variant filtering tree
chr10	*KIF11*	94366347	Exon-intron boundary-T rich	.	Deletion	AT	A	0.13	eliminated by variant filtering tree
chr11	*ACAT1*	108004927	Exon-intron boundary-T rich	.	Deletion	CT*	C	0.17	eliminated by variant filtering tree
chr11	*ACAT1*	108014662	Exon-intron boundary-T rich	rs5794587: benign variant	Insertion	T*	TA	0.7	eliminated by variant filtering tree
chr11	*PTS*	112099294	Exon-intron boundary-T rich	.	Deletion	CT*	C	0.21	eliminated by variant filtering tree
chr11	*SLC37A4*	118898435	splice junction	rs56966114: benign variant	Deletion	AC	A	1	eliminated by variant filtering tree
chr12	*WNK1*	987533	Exon-intron boundary-T rich	.	Insertion	C	CT	0.78	eliminated by variant filtering tree
chr13	*BRCA2*	32907535	Exon-intron boundary-T rich	.	Deletion	CT	C	0.14	eliminated by variant filtering tree
chr15	*ETFA*	76588127	Exon-intron boundary-T rich	.	Deletion	GT*	G	0.3	eliminated by variant filtering tree
chr16	*ERCC4*	14016109	Exon-intron boundary-T rich	rs113434368: no clinical significance	Deletion	CT*	C	0.12	eliminated by variant filtering tree
chr18	*MYO5B*	47455980	Exon-intron boundary -A rich	.	Deletion	GA	G	0.78	eliminated by variant filtering tree

chr19	*STXBP2*	7706746	Exon-intron boundary	.	Insertion	A	AGCCC	0.14	eliminated by variant filtering tree
chr19	*STXBP2*	7706754	Exon-intron boundary	.	Deletion	CAG	C	0.3	eliminated by variant filtering tree
